# Primary pancreas NTRK-rearranged neoplasm harboring an EVT6::NTRK3 fusion with a sclerosing epithelioid fibrosarcoma morphology: a case report and comprehensive review of the literature

**DOI:** 10.3389/fonc.2025.1526281

**Published:** 2025-06-06

**Authors:** Xin Wu, Dujuan Li, Fangfang Fu, Lifei Lian

**Affiliations:** ^1^ Department of Pathology, People’s Hospital of Zhengzhou University, Zhengzhou, China; ^2^ Department of Pathology, Renmin Hospital of Wuhan University, Wuhan, China; ^3^ Department of Medical Imaging, Henan Provincial People’s Hospital & Zhengzhou University People’s Hospital, Zhengzhou, China; ^4^ Department of Neurology, Tongji Hospital, Tongji Medical College, Huazhong University of Science and Technology, Wuhan, China; ^5^ Hubei Key Laboratory of Neural Injury and Functional Reconstruction, Huazhong University of Science and Technology, Wuhan, China

**Keywords:** NTRK-rearranged spindle cell neoplasms, pancreas, sclerosing epithelioid fibrosarcoma-like pattern, NTRK rearrangement, EVT6::NTRK3, pan-TRK immunohistochemistry, case report

## Abstract

NTRK-rearranged spindle cell neoplasms (NTRK-RSCNs) are an emerging soft tissue tumor entity characterized by NTRK gene fusions, occurring predominantly in the extremities of children and young adults. The diagnosis of this tumor is challenging due to its nonspecific and highly variable morphology. Given the response to selective NTRK inhibitors, it remains critical to identify the rare cases occurring in the viscera of adults. Here, we report a 53-year-old woman who presented with a new abdominal mass of half a month’s duration. Magnetic resonance imaging (MRI) showed a mass localized in the body and tail of the pancreas, leading to a partial pancreatectomy. Histologically, the tumor showed that bland monomorphic spindle cells were arranged in single rows of lines along the collagen fiber, reminiscent of sclerosing epithelioid fibrosarcoma. Immunohistochemically, the spindle cells focally expressed CD34 and S100 but lacked SOX10, MUC-4, Desmin, CK, and STAT6 expression. The tumor also showed cytoplasmic reactivity for pan-tyrosine receptor kinase (pan-TRK). Fluorescence *in situ* hybridization (FISH) analysis of NTRK1/NTRK2/NTRK3 gene break-apart probes identified NTRK3 rearrangement. Subsequent next-generation sequencing revealed EVT6 exon4::NTRK3 exon14 fusion. After surgery, the patient received continuous treatment with larotrectinib for 22 months and was followed up for 22 months without any signs of recurrence or metastasis. To further understand the clinical features, pathology, treatment and prognosis of this tumor, we searched the literature using different combinations of keywords ultimately obtaining 164 cases of NTRK-RSCNs (including the present case). Of these cases, 97 (59.1%) occurred in viscera, and 67 (40.9%) in soft tissues. There may be differences in age, histomorphology, immunophenotype, genetics, and prognosis between visceral and soft tissue NTRK-RSCNs. Appropriate immunohistochemical workup, including CD34, S100, and pan-TRK, and molecular tests, are indispensable in identifying this entity.

## Introduction

NTRK-rearranged spindle cell neoplasms (NTRK-RSCNs) are an emerging entity in the 5th edition of the World Health Organization (WHO) Classification of Tumors of Soft Tissue and Bone in 2020. NTRK-RSCNs are a molecularly defined tumor (1) that embraces a wide clinical and pathological spectrum, ranging from low-grade neoplasms to highly aggressive sarcomas (2, 3). It shows a variable spectrum of overlapping morphologies, including lipofibromatosis-like, malignant peripheral nerve sheath tumor (MPNST)-like, myopericytoma/hemangiopericytoma(MPC/HPC)-like, or inflammatory myofibroblastic tumor (IMT)-like pattern (4–6). The tumor demonstrates variable CD34 and/or S100 immunohistochemical expression, frequently with co-expression of CD34 and S100. The diagnosis of this tumor is difficult due to the nonspecific and variable morphology and immunophenotype. The clinical course of the tumor varies from being indolent and locally aggressive to metastasis. The majority arise in the soft tissues of the extremities and trunk of children and young adults. It rarely occurs in the visceral organs such as the gastrointestinal tract (7), uterus (8, 9), and lung (10). Although visceral NTRK-RSCNs are rare overall, their accurate diagnosis remains critical and clinically significant, given their considerable response to selective NTRK inhibitors. We report herein an NTRK-RSCN case with a sclerosing epithelioid fibrosarcoma morphology arising primarily in the adult pancreas, further expanding the clinical and pathological spectrum of NTRK-RSCNs. In addition, we reviewed 163 cases of NTRK-RSCNs reported in the literature. We summarized and compared the clinicopathological and prognostic features between visceral and soft tissue NTRK-RSCNs to better understand this rare entity.

## Case description

### Clinical data

**Figure 1:**
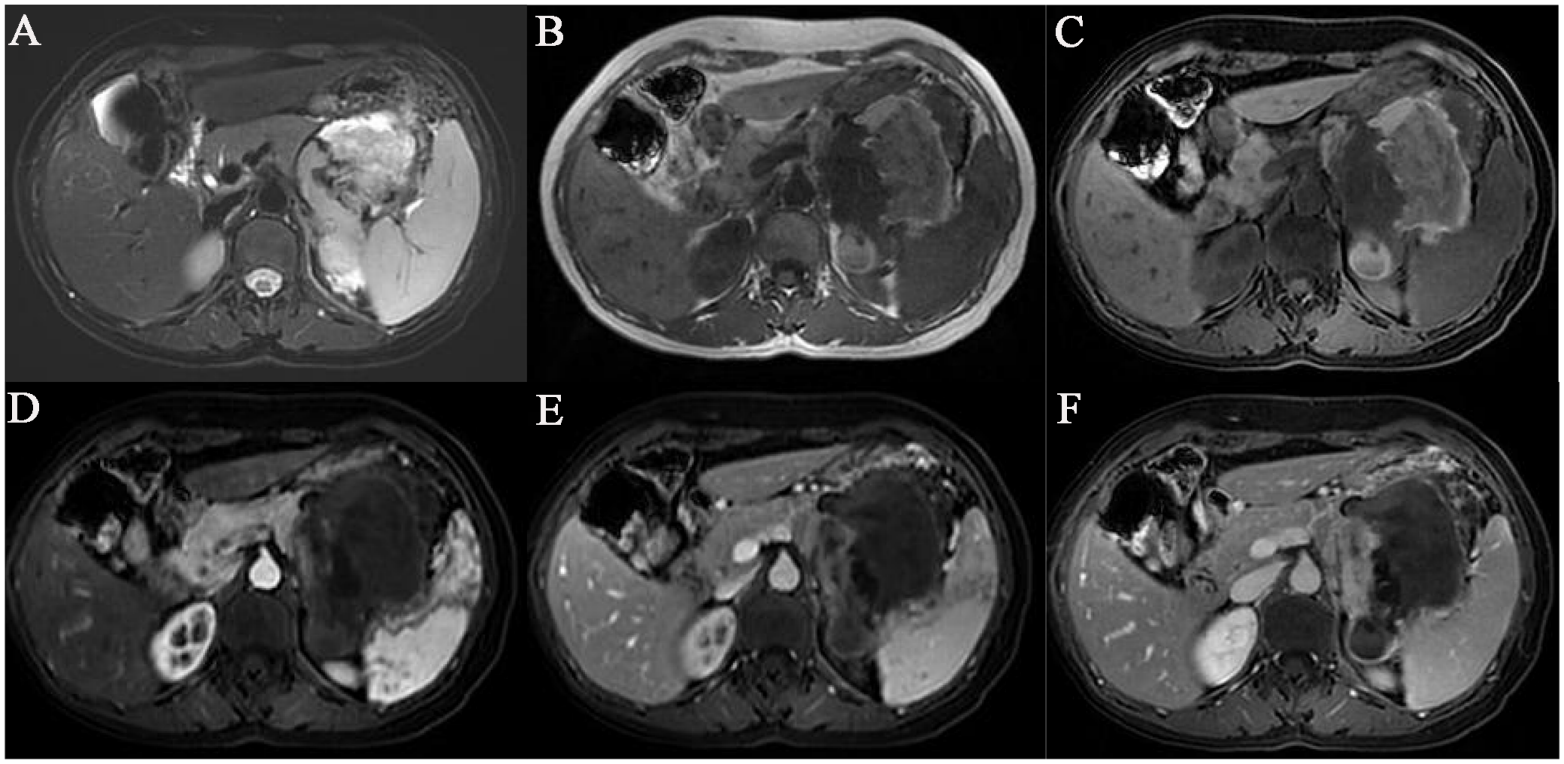
MRI showed an irregular mass of abnormal signal shadows in the tail of the body of the pancreas. **(A)** T2-weighted images fat suppression. **(B)** T1-weighted images. **(C)** T1-weighted images fat suppression. **(D)** Enhanced arterial phase. **(E)** Enhanced venous phase. **(F)** Enhanced delayed phase.

A 53-year-old female patient accidentally found a left upper abdominal mass on physical examination half a month ago. She was admitted to our hospital for further examination and treatment. The patient had no personal medical history or family history. Abdominal ultrasound showed heterogeneous echoes with unclear borders and irregular shapes in the body and tail of the pancreas. Magnetic resonance imaging (MRI) revealed an ill-defined and irregular mass in the body and tail of the pancreas. The lesion exhibited heterogeneous signals on T1-weighted and hyperintensity on T2-weighted images; the solid component of the mass showed inhomogeneous and delayed enhancement, with patches of non-significantly enhanced shadows seen inside enhancement scanning (**Figure 1**). MRI was suspicious of a solid pseudopapillary tumor of the pancreas. No obvious abnormality was found in the laboratory examination findings. The patient underwent surgery. Intraoperatively, a large mass of about 11.5 cm×8 cm×7.5 cm was seen in the body and tail of the pancreas, and the mass was found to be tightly adherent to the omentum, left adrenal gland, and spleen. The patient eventually underwent the resection of the body and tail of the pancreas, accompanied by omentectomy, left adrenalectomy, and splenectomy.

### Pathological examination

**Figure 2:**
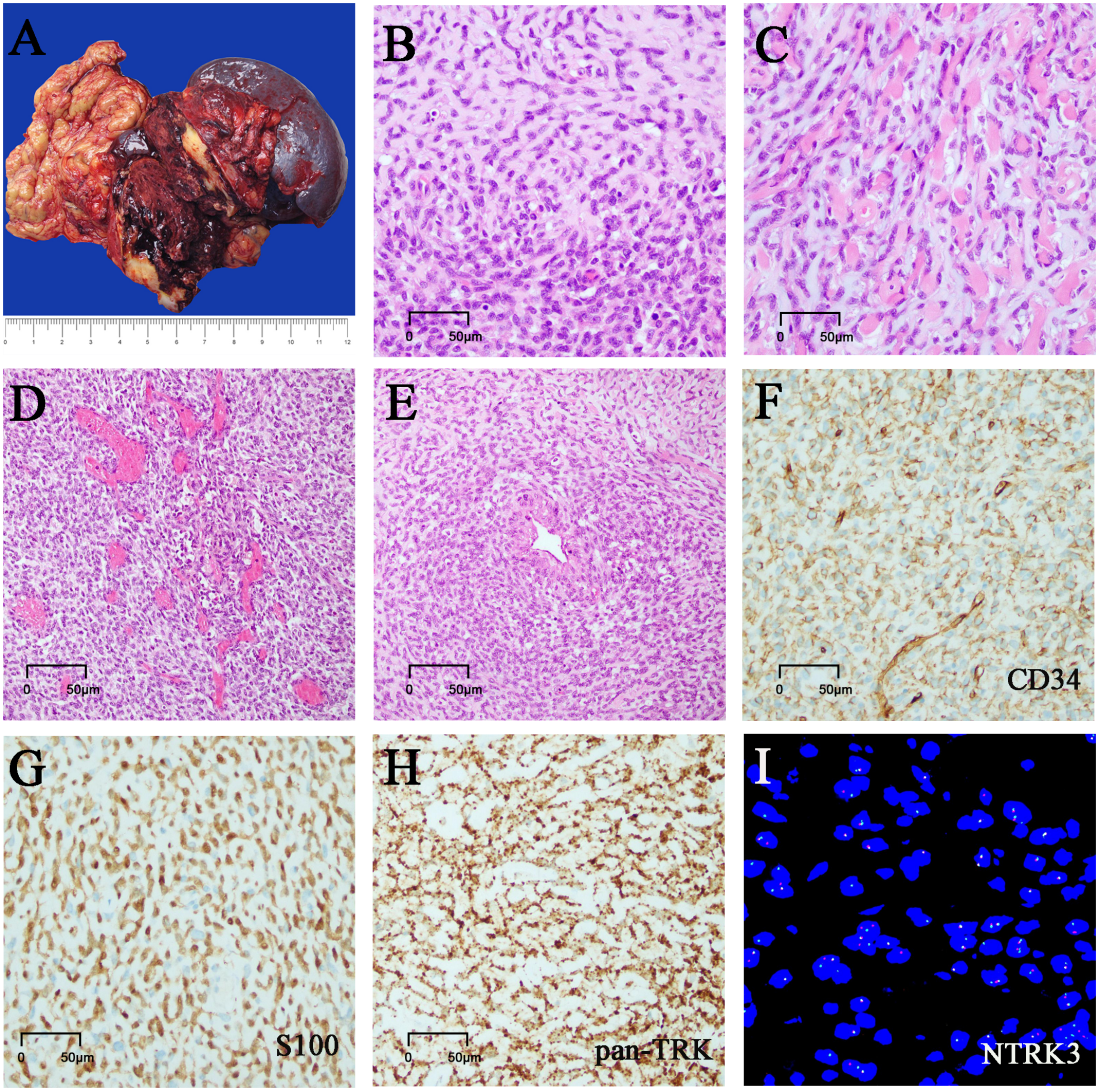
Morphology, immunostaining, and molecular tests. **(A)** Grossly, the mass is cystic and solid, and the cut surface is gray-white and gray-red. **(B)** Cell-rich zones and hypocellular zones are alternately distributed in tumors. **(C)** The short spindle cells were arranged in single rows of lines along the collagenous stroma with hyalinization and mild myxoedematous change. **(D)** Hemangiopericytoma-like staghorn vessels were also noted in focal areas. **(E)** Tumor cells were gathered around the perivascular. **(F)** Tumor cells showed focal staining of CD34. **(G)** Tumor cells showed focal staining of S100. **(H)** Tumor cells showed diffuse and strong staining of pan-TRK. **(I)** NTRK3 rearrangement tested by FISH analysis.

Gross pathological observation of the resection specimen showed the following: a large mass measuring 11.5 cm × 8 cm × 7.5 cm in the body and tail of the pancreas, partially adhering to the omental tissue, and the cut surface of the mass being cystic solid, with soft-medium texture, and appearing grayish red, dark red, grayish white (**Figure 2A**); attached omentum measuring 18.5 cm× 9.5 cm × 2.5 cm; attached spleen measuring 17.5 cm × 8.5 cm × 7.5 cm; attached left adrenal gland measuring 4.5 cm × 4.2 cm × 1cm.

Microscopically, the tumor was generally well circumscribed but unencapsulated, and focal infiltration of the peripancreatic adipose and the omental tissue was present. It was comprised of monomorphic short spindle and oval cells with low to focal high cellularity (**Figure 2B**). The short spindle cells were arranged in single rows of lines along the collagenous stroma with hyalinization and mild myxoedematous change, resembling sclerosing epithelioid fibrosarcoma (**Figure 2C**). The tumor cells had scarce or a small amount of eosinophilic cytoplasm, with ovoid to spindle nuclei containing fine chromatin and indistinct nucleoli. The nuclear atypia was mild. The tumor showed variable mitotic activity, ranging from 1 to 15 mitoses per 10 high-power fields (HPFs). No tumor necrosis was found. There were marked vessels characterized by ring-like perivascular hyalinization in low-moderate cellularity areas and some curved or antler-shaped thin-walled blood vessels in high cellularity areas (**Figure 2D**). Focal tumor cells were densely distributed around the endothelium and blood vessels (**Figure 2E**). A few scattered foamy histiocytes, hemosiderin cells, lymphocytes, and multinucleated giant cells were noted in focal areas. Some areas showed marked hemorrhage and degenerative changes. A few benign pancreatic ducts entrapped were present within the tumor.

Immunohistochemically, tumor cells showed patchy staining of CD34 and S100 protein (**Figures 2F, G**), with weak focal staining for α- SMA, calponin, and Bcl-2. They were negative for SOX-10, STAT6, AE1/AE3, EMA, CK7, CK8/18, CK5/6, CD117, DOG-1, desmin, MUC4, CD99, CDK4, CD31, ERG, HMB-45, melan A, ALK, CR, CD68. The tumor cells showed strong, diffuse cytoplasmic staining for pan-TRK monoclonal antibody (EPR17341, Roche; **Figure 2H**). Immunostaining of H3K27Me3, INI-1, BRG-1, and RB was retained, and β-catenin showed cytoplasmic positivity. Ki67 index was 5% ~ 15%.

Fluorescence *in situ* hybridization (FISH) analysis of NTRK1/NTRK2/NTRK3 gene break-apart probes identified NTRK3 gene rearrangement (**Figure 2I**). Subsequent analysis of next-generation sequencing (NGS) demonstrated an EVT6 exon 4::NTRK3 exon14 fusion. In addition, CDKN2A/2B homozygous deletion and ARID1A mutation were also identified.

### Treatment and follow-up

After surgery, the patient received continuous treatment with regular oral NTRK inhibitor - larotrectinib (100mg twice a day) for 22 months and was followed up for 22 months without any signs of recurrence or metastasis.

### Literature review

#### Methodology

An extensive literature search was performed to identify previously reported cases of NTRK-RSCNS in PubMed (http://www.ncbi.nlm.nih.gov/pubmed/) using different combinations of keywords in the title/abstract field, including “NTRK-rearranged spindle cell neoplasm”, “NTRK-rearranged neoplasm”, “NTRK fusion”, “pan-TRK immunohistochemistry”, “NTRK1”, “NTRK2”, “NTRK3” until July 2024. Cases in the English literature were reviewed to extract the various essential clinicopathological data, including clinical presentation, histological features, immunohistochemical phenotypes, molecular genetics, management approaches, and outcomes. Data from redundant cases in separate papers were combined. We only included cases that fulfilled the criteria of NTRK-RSCNs as defined by the 5th edition of the WHO Classification of Tumors of Soft Tissue and Bone in 2020.

## Results

### Clinical features of NTRK-RSCNs

A total of 164 cases were identified (163 from previous literature and one from the present case). The 164 cases occurred in 111 females and 53 males with ages ranging from 1 month to 77 years (median, 30 years). Of these cases, 97 patients (59.1%) occurred in the viscera, involving the uterus (50 cases) (5, 8, 9, 11–27), gastrointestinal tract (28 cases) (4, 5, 7, 28–33), lung (13 cases) (5, 10, 19, 34–37), liver (2 cases) (19, 37), prostate (1 case) (5), heart (1 case) (5), brain (1 case) (17) and pancreas (1 case). Sixty-seven cases (40.9%) arose in the soft tissue, including the limbs (34 cases) (2, 4, 17, 24, 30, 33, 36, 38–41), trunk (22 cases) (2, 4, 17, 24, 30, 33, 41–45), and head and neck (11 cases) (2, 17, 19, 30, 36, 41, 42, 46). The maximum diameter of the masses ranged from 0.6 cm to 25 cm (median: 5.2 cm; mean: 6.8 cm) based on radiologic and/or gross examinations. Clinically or radiologically, most cases were described as ill-defined or infiltrative tumors. The brief clinical characteristics of 97 patients with visceral NTRK-RSCNs were summarized in **Table 1**.

**Table 1:** Clinical and prognostic data of 97 cases of visceral NTRK-RSCNs.

Case	Age	Sex	Site	Size (cm)	CD34	S100	Pan-TRK	Gene fusion	Follow-up(mo)	Source
1	2mo	M	stomach	7	+	–	d	TPM3-NTRK1	LTFU	Atiq et al. (28)
2	4mo	F	intestine	3	–	–	d	EVT6-NTRK3	LTFU	Atiq et al. (28)
3	5mo	M	intestine	12.5	–	–	d	EVT6-NTRK3	DOD (2)	Atiq et al. (28)
4	3y	M	Mesentery	NA	–	–	d	SPECC1L-NTRK3	LTFU	Atiq et al. (28)
5	3y	F	intestine	NA	+	+	d	NA	NA	Yin et al. (4)
6	4y	F	stomach	7	+	+	d	LMNA-NTRK1	LTFU	Atiq et al. (28)
7	6y	F	rectum	6	–	–	d	TPM3-NTRK1	NED (13)	Gao et al. (7)
8	7y	M	descending colon	4.5	+	+	d	LMNA-NTRK1	NED (6)	Gao et al. (7)
9	7y	F	lung	4.5	NA	NA	NA	NA	NED (96)	Yamamoto et al. (35)
10	7y	M	intestine	8.5	+	+	d	TPR-NTRK1	NED (3.5)	Atiq et al. (28)
11	11y	M	Mesentery	11	+	+	d	NTRK3 gene rearrangement	DOD (1)	Suurmeijer et al. (33)
12	13y	F	Cervix	9.2	+	+	d	TPM3-NTRK1	NED (4)	Goulding et al. (11)
13	15y	M	lung	NA	+	+	d	BPMS-NTRK3	NED (4)	Brčić et al. (36)
14	16y	F	Cervix	NA	+	+	d	TPR-NTRK1	NED (30)	Costigan et al. (9)
15	17y	M	stomach	5	+	+	d	TPM3-NTRK1	NED (14)	Gao et al. (7)
16	17y	F	lung	19	+	+	NA	EVT6-NTRK3	NA	Alassiri et al. (37)
17	17y	M	brain	NA	+	+	NA	TPM3-NTRK1	AWD (13)	Tauziède-Espariat et al. (17)
18	18y	M	prostate gland	2	+	+	d	TPM3-NTRK1	NED (34)	Tsai et al. (5)
19	18y	M	stomach	NA	NA	NA	NA	LMNA-NTRK1	DOD (25)	Suurmeijer et al. (30)
20	18y	F	right pleura	13.5	+	+	d	TPM3-NTRK1	NA	Tsai et al. (5)
21	23y	F	Cervix	3	+	+	d	TPM3-NTRK1	NED (33)	Croce et al. (12)
22	23y	M	lung	2	NA	NA	NA	EVT6-NTRK3	NED (121)	Yamamoto et al. (35)
23	23y	M	Rectum	4	+	+	d	LMNA-NTRK1	NA	Yin et al. (4)
24	24y	F	Cervix	15	–	+	d	SPECC1L-NTRK3	AWD (52)	Rabban et al. (13)
25	24y	F	Cervix	NA	+	+	d	TPM3-NTRK1	NED (16.5)	Costigan et al. (9)
26	24y	F	Cervix	2.2	+	+	f	TPM3-NTRK1	NED (36M)	Bühler et al. (14)
27	25y	M	Rectum	NA	–	–	NA	NTRK1 gene rearrangement	AWD (25)	Gao et al. (7)
28	25y	F	ascending colon	2.5	+	+	NA	TPM3-NTRK1	NA	Atiq et al. (28)
29	26y	F	Cervix	12	+	+	d	EML4-NTRK3	AWD (52)	Croce et al. (12)
30	26y	F	Cervix	12	+	+	f	EML4-NTRK3	NED (82)	Costigan et al. (9)
31	26y	F	Cervix	5.5	–	–	d	TFG-NTRK3	DOD (22)	Costigan et al. (9)
32	26y	F	Cervix	8	–	+	d	TPM3-NTRK1	LTFU	Costigan et al. (9)
33	26y	F	corpus	23	–	+	d	STRN-NTRK3	NED (36)	Michal et al. (15)
34	26y	F	Cervix	NA	–	+	f	NA	DOD (84)	Costigan et al. (9)
35	26y	F	Cervix	22.7	+	+	d	STRN-NTRK3	NA	Klubickova et el. (24)
36	27y	F	corpus	16.3	–	+	d	LMNA-NTRK1	NED (11)	Chiang et al. (16)
37	29y	F	Cervix	NA	+	+	NA	TPM3-NTRK1	NED (11)	Tauziède-Espariat et al. (17)
38	30y	F	Cervix	2.5	+	+	d	TPM3-NTRK1	NED (12)	Croce et al. (12)
39	30y	F	Cervix	2.5	+	+	NA	TPM3-NTRK1	NED (4)	Wells et al. (18)
40	30y	F	Cervix	2.5	NA	–	d	TPM3-NTRK1	AWD (37)	Costigan et al. (9)
41	31y	F	Cervix	9	+	+	f	NTRK3 gene rearrangement	LTFU	Wong et al. (19)
42	31y	M	lung	1.7	+	+	d	TPM3-NTRK1	NED (4)	Zhu et al. (10)
43	31y	M	lung	1.8	NA	NA	NA	EVT6-NTRK3	NED (6)	Chang et el (34).
44	31y	M	lung	1.8	+	+	d	TPM3-NTRK1	NED (9)	Zhu et el (10).
45	32y	M	lung	1.8	+	+	d	TPM3-NTRK1	NED (9)	Zhu et al. (10)
46	32y	F	Cervix	8	+	+	d	TPR-NTRK1	Recurrence (8)	Grant et el (26).
47	33y	F	corpus	5	+	+	d	TPM3-NTRK1	NED (108)	Croce et al. (12)
48	34y	F	Cervix	14.6	+	+	d	SPECC1L-NTRK3	AWD (40), lung Metastasis	Grant et el (26).
49	34y	F	Ascending colon	6.5	+	–	NA	LMNA-NTRK1	NED (4)	Gao et al. (7)
50	34y	M	lung	7	–	+	d	LMNA-NTRK1	AWD (5)	Tsai et al. (5)
51	34y	F	Rectum	3	+	+	d	IGR (downstream PMVK)-NTRK1	NED (12)	Yin et al. (4)
52	35y	F	corpus	9.4	NA	NA	NA	C16orf72-NTRK1	LTFU	Costigan et al. (9)
53	35y	F	Cervix	3.5	+	+	d	TPM3-NTRK1	LTFU	Costigan et al. (9)
54	37y	F	Cervix	6.3	+	+	d	IRF2BP2-NTRK1	LTFU	Devereaux et al. (20)
55	38y	M	liver	20	NA	NA	NA	EVT6-NTRK3	NA	Alassiri et al. (37)
56	39y	F	Cervix	NA	+	+	d	TPM3-NTRK1	LTFU	Croce et al. (12)
57	39y	F	Cervix	5.8	+	+	–	TPM3-NTRK1	NED (9)	Devereaux et al. (20)
58	39y	F	Cervix	NA	+	+	NA	TPM3-NTRK1	LTFU	Costigan et al. (9)
59	40y	F	Cervix	2	+	+	f	TPR-NTRK1	NED (32)	Devereaux et al. (20)
60	42y	F	Cervix	2.6	–	+	d	TPR-NTRK1	NED (2)	Chiang et al. (16)
61	42y	F	Cervix	NA	–	+	d	NA	LTFU	Costigan et al. (9)
62	42y	F	Cervix	5.2	+	–	d	TPM3-NTRK1	NED (11)	Boyle et al. (21)
63	42y	F	Cervix	5.6	–	+	d	TPR-NTRK1	NED (44)	Costigan et al. (9)
64	42y	F	Cervix	5.2	–	–	d	TPM3-NTRK1	Metastasis(29)	Grant et el (26).
65	43y	F	Transverse colon	3.7	+	–	d	LMNA-NTRK1	NED (12)	Gao et al. (7)
66	43y	F	Cervix	9.4	+	+	d	EML4-NTRK3	NED (6)	de Castro et el (27).
67	43y	F	Cervix	8	+	–	d	NUMA1-NTRK1	Recurrence (2)	Szalai et el (25).
68	44y	M	Small intestine	9	+	+	NA	NTRK1 gene rearrangement	DOD (12)	Gao et al. (7)
69	44y	F	Cervix	4.5	+	+	d	TPM3-NTRK1	NED (2)	Croce et al. (12)
70	44y	M	Rectum	5	NA	NA	NA	EVT6-NTRK3	NED (44)	Brenca et al. (29)
71	44y	F	Rectum	11	–	–	d	TPM3-NTRK1	AWD (50)	Atiq et al. (28)
72	45y	F	lung	1.2	+	+	d	LMNA-NTRK1	NED (87)	Zhu et al. (10)
73	46y	F	Cervix	9.3	–	+	d	TPM3-NTRK1	AWD (7)	Chiang et al. (16)
74	46y	F	Cervix	10	–	NA	d	IRF2BP2-NTRK1	LTFU	Costigan et al. (9)
75	47y	F	Cervix	14	–	+	d	RBPMS-NTRK3	DOD (79)	Chiang et al. (16)
76	47y	F	Cervix	2.7	+	+	f	TPM3-NTRK1	Recurrence and Metastasis (21)	Tsai et al. (5)
77	47y	F	Cervix	7.8	NA	NA	d	TPR-NTRK1	DOD (12)	Costigan et al. (9)
78	48y	F	Left ventricle	4.5	+	+	d	SQSTM1-NTRK3	AWD (7)	Tsai et al. (5)
79	49y	F	Cervix	1.8和1.4	–	+	d	TPR-NTRK1	NED (≥6)	Rabban et al. (13)
80	49y	F	Mesentery	25	+	+	f	NTRK3 gene rearrangement	AWD (40), liver Metastasis	Tsai et al. (5)
81	52y	F	Cervix	1.3	+	+	NA	TPM3-NTRK1	NED (6)	Nilforoushan et al. (8)
82	53y	F	Cervix	6.8	+	–	d	TPM3-NTRK1	AWD (9)	Tsai et al. (5)
83	53y	F	pancreas	11.5	+	+	d	EVT6-NTRK3	NED (22)	This case
84	54y	F	Cervix	5.4	+	+	NA	SPECC1L-NTRK3	Recurrence (8)	Nilforoushan et al. (8)
85	54y	M	colon	NA	NA	NA	NA	EVT6-NTRK3	NA	Shi et al. (31)
86	55y	F	ascending colon	NA	+	+	NA	LMNA-NTRK1	NA	Gao et al. (7)
87	55y	M	intestine	NA	NA	NA	NA	EVT6-NTRK3	NA	Shi et al. (31)
88	55y	M	intestine	10.1	+	+	d	TPM3-NTRK1	NED (5)	Atiq et al. (28)
89	55y	F	Cervix	1.6	+	+	NA	SPECC1L-NTRK3	NED (8)	Hodgson et al. (22)
90	59y	M	abdomen	NA	NA	NA	NA	EVT6-NTRK3	NA	Castillon et al. (32)
91	61y	F	lung	1.1	NA	NA	NA	EVT6-NTRK3	Recurrence (1)	Chang et al. (34)
92	61y	F	Cervix	7	+	+	d	SPECC1L-NTRK3	NED (16)	Costigan et al. (9)
93	62y	M	liver	NA	NA	+	NA	EVT6-NTRK3	NA	Wong et al. (19)
94	63y	M	Duodenum	5	–	+	d	STRN-NTRK2	NED (30)	Gao et al. (7)
95	65y	F	lung	0.6	–	+	d	EVT6-NTRK3	NA	Wong et al. (19)
96	66y	F	Cervix	1.5	+	+	d	TPM3-NTRK1	NED (2)	Devereaux et al. (20)
97	69y	F	Cervix	7	NA	NA	NA	WWOX-NTRK2	NA	Moh et al. (23)

mo, months; y, years old; F, female; M, male; +, positive; -, negative, d, diffuse; f, focal; NA, not available; DOD, dead of disease; NED, no evidence of disease; AWD, alive with disease; LTFU, lost to follow up.

### Histologic characteristics of NTRK-RSCNs

Histologically, the tumor borders were at least focally infiltrative in almost all cases. The tumors were mainly composed of spindle cells and focally epithelioid cells, with widely varying cellularity and haphazard to the fascicular arrangement. Prominent collagen deposition, keloidal collagen fibers, and stromal/perivascular hyalinization were frequently evident in these cases. Myxoedematous stromal change, staghorn vessels, and lymphocytic infiltrates were also commonly found. The mitotic activity was highly variable. Tumor necrosis was often identified in cases with high-grade histology. These cases generally formed a low to high-grade tumor spectrum and exhibited various growth patterns. The low-grade cases exhibited mild nuclear atypia and relatively low cellularity (28/73, 38.4%), resembling LPF-NT (21/73, 28.8%) (2, 4, 25, 36, 41, 46), IMT (4/73, 5.5%) (35, 37), MPC/HPC (2/73, 2.7%) (42), dermatofibrosarcoma protuberans (DFSP) (1/73, 1.4%) (43). The high-grade cases demonstrated moderate to high cellularity (44/73, 60.2%), resembling MPNST/fibrosarcoma (40/73, 54.8%) (4, 5, 7, 17–19, 25, 28, 33, 36, 38), myxofibrosarcoma(2/73, 2.7%) (39, 42)and adenosarcoma(2/73, 2.7%) (5). Notably, we reported for the first time an extremely rare and unique morphology of NTRK-RSCN resembling a sclerosing epithelioid fibrosarcoma (**Figures 2B–E**). Very few cases had focal rhabdomyoblast and pleomorphic liposarcomatous differentiation (5).

### Immunohistochemical characteristics of NTRK-RSCNs

The results of immunohistochemistry of NTRK-RSCNs were summarized in **Table 2**. Variable CD34 and S100 were expressed in 79.5% (101/127) and 81.9% (104/127) of cases, respectively. Of 127 cases, 69.3% of cases (88/127) co-expressed CD34 and S100. Of note, about 31% of cases showed only one positive expression or both negative expression of CD34 and S100, including CD34-/S100+ (12.6%), CD34+/S100- (10.2%), or CD34-/S100- (7.9%). Generally, CD34 and S100 were diffusely expressed in low-grade tumors, whereas they were often focally expressed in high-grade tumors. The pan-TRK immunohistochemical data of 102 patients are available. Variable extent expression of pan-TRK was noted in almost all cases (101/102, 99%), except for 1 case (1%, 1/102) (20). Diffuse and focal positive expression of pan-TRK accounted for 85.3% and 13.7%, respectively. Pan-TRK was mainly expressed in the cytoplasm of tumor cells, with very few cases expressed simultaneously in the cytoplasm and nucleus. All cases were negative for AE1/AE3, CD117, DOG1, SOX-10, STAT6, desmin, and ALK. Focal staining for SMA and calponin was positive in a few cases (9, 16, 17, 19, 22, 28, 37–40, 42). The expression of p16 was completely lost in some cases (5, 8, 26). The expression of p53 showed a mutant (null) pattern in the heterologous sarcomatous transformation (5). H3K27me3 staining was retained in all cases except one (43). Ki67 index ranged from 10% to 50%.

**Table 2:** Clinicopathological characterization of 164 patients with NTRK-RSCNs.

Characteristic	Viscera	Soft tissue	*P* value
Location	uterus (51.6%, 50/97) gastrointestinal tract (28.9%, 28/97) lung (13.4%, 13/97) liver (2.1%, 2/97) prostate gland (1%, 1/97) heart (1%, 1/97) brain (1%, 1/97) pancreas (1%, 1/97)	limbs (50.8%, 34/67) trunk (32.8%, 22/67) head and neck (16.4%, 11/67)	
Sex			
M	(26.8%, 26/97)	(40.3%, 27/67)	0.069
F	(73.2%, 71/97)	(59.7%, 40/67)	
Age, M(R)	34 (2mo, 69y)	21 (1mo, 77y)	0.001
≤21	20	33	0.000
>21	77	34	
Histological patterns			
LPF-NT-like	0	21	
MPNST/fibrosarcoma-like	21	19	
myxofibrosarcoma-like	0	2	
DFSP-like	0	1	
MPC/HPC-like	0	2	
adenosarcoma-like	2	0	
IMT-like	4	0	
sclerosing epithelioid fibrosarcoma-like	1	0	
Immunohistochemistry			
CD34(+)	58 (73.4%)	43 (89.6%)	0.009
S100(+)	66 (83.5%)	38 (79.2%)	0.208
CD34(+)/S100(+)	52 (65.8%)	36 (75%)	0.227
CD34(+)/S100(-)	6 (7.6%)	7 (14.6%)	0.237
CD34(-)/S100(+)	14 (17.7%)	2 (4.2%)	0.026
CD34(-)/S100(-)	7 (8.9%)	3 (6.2%)	0.741
Pan-TRK			
diffuse	62 (87.3%)	25 (80.6%)	0.379
focal	8 (11.3%)	6 (19.4%)	0.349
negative	1 (1.4%)	0	
NTRK rearrangements			
NTRK1	58 (61.7%)	53 (79.1%)	0.019
TPM3-NTRK1	33 (58.9%)	14(33.3%)	
LMNA-NTRK1	10 (12.8%)	19 (38.8%)	
TPR-NTRK1	7 (17.9%)	3 (7.1%)	
IRF2BP2-NTRK1	2	2	
TMB3-NTRK1	0	1	
GAS2L1-NTRK1	0	1	
NUMA1-NTRK1	1	0	
C16orf72-NTRK1	1	0	
IGR (downstream-PMVK)-NTRK1	1	0	
NTRK3	34 (36.2%)	13(19.4%)	0.021
EVT6-NTRK3	16 (51.6%)	1	
SPECC1L-NTRK3	6 (19.4%)	0	
STRN-NTRK3	2	2	
EML4-NTRK3	3	2	
TFG-NTRK3	1	2	
SQSTM1-NTRK3	1	1	
BPMS-NTRK3	1	0	
RBPMS-NTRK3	1	1	
TPM4-NTRK3	0	1	
NTRK2	2(2.1%)	1 (1.5%)	1.000
SPECC1L-NTRK2	0	1	
STRN-NTRK2	1	0	
WWOX-NTRK2	1	0	
Genomic co-alterations			
CDKN2A/2B deletion	12	12	
SMAD4 deletion	0	1	
CHEK2 deletion	0	1	
FOXL2 mutation	1	0	
ARID1A mutation	1	0	
MCL1 Copy number increase	0	2	
MYC Copy number increase	0	1	
Treatment			
surgical resection	71	59	
surgical resection, radiotherapy	12	5	
Surgical resection and targeted therapy	6	0	
Surgical resection, radiotherapy, targeted therapy	2	1	
Follow-up(2~648months)			
alive	63	46	
NED	45	34	
alive with recurrence/metastasis	18	12	
dead	9	4	

M(R), Median (Range); mo, months; y, years; M, Male; F, Female; +, positive; -, negative; LPF-NT, lipofibromatosis-like neural tumor; MPNST, malignant peripheral nerve sheath tumor; MPC/HPC, myopericytic/haemangiopericytic pattern; IMT, inflammatory myofibroblasts; DFSP, dermatofibrosarcoma protuberans; focal, < 50% of tumor cells are stained; diffuse, > 50% of tumor cells are stained; N, negative; NED, no evidence of disease. P-values are calculated by the Wilcox test or Fisher test among visceral and soft tissue NTRK-RSCNs, P < 0.05 represented the significant difference.

### Molecular genetics of NTRK-RSCNs

Among the 164 cases of NTRK-RSCNs, molecular cytogenetic data of 161 cases were available and were summarized in **Table 2**. Of the 161 cases, 111 cases (68.9%) were positive for NTRK1 rearrangement, 47 cases (29.2%) were positive for NTRK3 rearrangement, and 3 cases (1.9%) were positive for NTRK2 rearrangement. NTRK1 genes was rearranged with a significant number of partner genes, including TMP3 (47 cases), LMNA (29 cases), TPR (10 cases), IRF2BP2 (4 cases), TMB3 (1 case), GAS2L1 (1 case), NUMA1 (1 case), C16orf72 (1 case), and IGR (downstream-PMVK) (1 case). Partner genes for NTRK3 genes included ETV6 (17 cases), SPECC1L (6 cases), STRN (4 cases), EML4 (5 cases), TFG (3 cases), SQSTM1(2 cases), RBPMS (2 cases), BPMS (1 case), and TPM4 (1 case). The partner genes for NTRK2 genes were SPECC1L (1 case), WWOX (1 case), and STRN (1 case). In addition, co-occurrence of NTRK gene fusion with other oncogenic gene alterations was detected in 31 cases (19.3%). Copy number reduction of CDKN2A/2B (24 cases, 77.4%) was the most common change (5, 9, 12, 17, 24, 38, 42, 44). 14/24 cases had definite histologic patterns, with 4 cases of low-grade patterns (2 LPF-NT-like and 2 MPC/HPC-like) and 10 cases of high-grade patterns (7 MPNST/fibrosarcoma-like, 1 myxofibrosarcoma-like, 1 adenosarcoma-like and 1 sclerosing epithelioid fibrosarcoma-like). According to the above results, we prefer to consider that CDKN2A/2B copy number deletion is associated with high-grade histological morphology. Others included copy number reduction of SMAD4 (1 case) and CHEK2 (1 case), copy number increase of MCL1 (2 cases) and MYC (1 case) (4), mutations in FOXL2 (1 case) (4) and ARID1A (1 case).

### Treatment and follow-up of NTRK-RSCNs

One hundred and fifty-six patients underwent mass resection. A minority of these patients (16.7%, 26/156) were supplemented with radiotherapy and/or targeted therapy. Follow-up information was available for 122 of 164 patients (78.2%). The follow-up duration ranged from 2 to 648 months. 30 of 122 patients (26.1%) developed local recurrence or metastasis. The duration of recurrence/metastasis ranged from 1 to 60 months.

### Comparison of the clinicopathological features between visceral and soft tissue NTRK-RSCNs

A comparison of clinicopathological and prognostic features between visceral and soft tissue NTRK-RSCNs was shown in **Table 2**. Among the 164 reported cases, the proportions of NTRK rearranged spindle cell tumors occurring in viscera and soft tissues were 59.1% and 40.9%, respectively. The age range of NTRK-RSCNs in soft tissue and visceral organs was 2mo~69 y (median age of 34 years) and 1mo~77y (median age of 21 years), respectively. A significant difference was observed between age stratification (<21 years vs. ≥21 years) and the anatomical distribution of NTRK-RSCNs (visceral vs. soft tissue) (*P*=0.000). Patients aged ≥21 years showed a higher incidence of visceral NTRK-RSCNs, whereas those under 21 years had a higher prevalence of soft tissue NTRK-RSCNs. Histomorphologically, the LPF-NT-like (46.7%, 21/45) and MPNST/fibrosarcoma-like (42.2%, 19/45) patterns were frequently reported in the cases of soft tissue NTRK-RSCNs, suggesting that both low-grade and high-grade morphology are common in the soft tissue cases. In contrast, the low-grade morphology was not reported in the visceral cases. Seventy-four percent of the viscera cases (21/28) showed the MPNST/fibrosarcoma-like pattern. Notably, the myxofibrosarcoma-like (2/45), DFSP-like (1/45) and MPC/HPC-like (2/45) patterns were only reported in the cases of soft tissue NTRK-RSCNs. In contrast, the adenosarcoma-like (2/28), inflammatory myofibroblastoma-like (4/28) and sclerosing epithelioid fibrosarcoma-like (1/28) pattern were only reported in the viscera cases. Thus, there may be differences in the histomorphology between visceral and soft tissue NTRK-RSCNs. Immunohistochemically, the CD34 positive expression was higher in soft tissue than in viscera NTRK-RSCNs (89.6% vs. 73.4%; *P*=0.009). The positive expression rates of S100 in the soft tissue and viscera cases were 79.2% and 83.5%, respectively, with no difference between the two groups of cases. The expression of CD34-/S100+ was higher in the viscera NTRK-RSCNs than that of soft tissue NTRK-RSCNs (17.7% vs. 4.2%; *P*=0.026). There was no difference in the expression of CD34+/S100+ (65.8% vs. 75%; *P*=0.227), CD34-/S100+ (7.6% vs. 14.6%; *P*=0.237), or CD34-/S100- (8.9% vs. 6.2%; P=0.741) between the two groups. Pan-TRK immunohistochemistry showed variable positive expression in all soft tissue cases and the visceral cases except for one case. NTRK1 gene fusion rate was higher in soft tissues than that in viscera (79.1% vs. 61.7%, *P*=0.019), while the NTRK3 gene fusion rate was higher in viscera than that in soft tissues (36.2% vs. 19.4%, *P*=0.021). NTRK2 gene fusion was rare in 2 visceral cases and 1 soft tissue case. CDKN2A/2B deletion was reported in 12 visceral cases and 12 soft tissue cases.

## Discussion

Neurotrophic receptor tyrosine kinase genes, including NTRK1, NTRK2, and NTRK3, encode members of the tropomyosin receptor kinase (Trk) family, which include three transmembrane protein receptors TrkA, TrkB, and TrkC. These maintain the development and maintenance of the neuronal system through the activation of downstream pathways such as the RAS/MAPK, PI3K/AKT, and PLC-γ pathways (47, 48). NTRK fusions lead to constitutive activation or overexpression of Trk receptors, promoting the occurrence of tumors. NTRK fusion was first discovered in colon carcinoma in 1982 (49). The recognition and accurate diagnosis of NTRK fusion-positive cancers is critical to the treatment of patients because TRK inhibitors have high remission rates (>75%) regardless of tumor type (50). NTRK-RSCNs are an emerging entity included in the latest WHO classification molecularly characterized by NTRK rearrangements. We conducted a comprehensive review of reported isolated cases and series and obtained a total of 164 cases. Hence, the current study represents the largest series analyzing clinicopathologic characteristics of patients with NTRK-RSCNs.

NTRK-RSCNs spanned a wide range of ages, ranging from 1 month to 77 years (median: 30 years; mean: 30.6 years). Of note, soft issue NTRK-RSCNs predominantly occurred in children and adolescents (median: 21 years), whereas visceral NTRK-RSCNs preferentially occurred in young and middle-aged adults (median: 34 years). The cohort had a significant female predilection (2.2:1). Tumor size ranged from 0.6 to 25 cm (median: 5.2cm, mean: 6.8 cm). Of 164 cases, 40.9% of NTRK-RSCNs occurred in the superficial and deep soft tissues of the extremities, trunk or head and neck, while 59.1% of NTRK-RSCNs occurred in the viscera. The tumors occurred predominantly in the uterus (50/97, 51.6%), followed by the gastrointestinal tract and lungs, and occasionally in the liver, heart, prostate, and brain. Herein, we report the first case of primary pancreas NTRK-RSCN. This suggests that the visceral NTRK-RSCNs are not so rare and may be missed or misdiagnosed. For spindle cell tumors that occur in visceral organs, the possibility of the NTRK-RSCNs also needs to be considered.

NTRK-RSCNs have various morphological patterns and tumor grades. Low-grade lesions displayed low cellularity and low mitotic activity and are characterized by distinctive stromal collagen deposition and perivascular band-like hyalinization. The reported low-grade morphology includes LPF-NT-like, dermatofibrosarcoma protuberans-like, myopericytic/haemangiopericytic or inflammatory myofibroblast-like appearance. High-grade lesions were consistent with high-grade sarcomas, displaying cytological atypia with brisk mitotic activity (>10/10HPF), which primarily included MPNST, myxofibrosarcoma-like, and adenosarcoma-like and sclerosing epithelioid fibrosarcoma-like morphology. Based on our comprehensive review of the reported cases, the soft tissue NTRK-RSCNs showed both low-grade and high-grade morphology, including LPF-NT-like and MPNST/fibrosarcoma-like patterns, as well as rare mucinous fibrosarcoma-like, DFSP-like and MPC/HPC-like morphologies. Visceral NTRK-RSCNs mainly exhibited high-grade MPNST/fibrosarcoma-like patterns, while LPF-NT morphologies have not been reported. In viscera, there were also some different morphologies from those in soft tissues, including adenosarcoma-like, inflammatory myofibroblastoma-like, and, in this case, sclerosing epithelioid fibrosarcoma-like pattern. Thus, there may be differences in the histomorphology between visceral and soft tissue NTRK-RSCNs. Herein, we reported an EVT6-NTRK3 fusion-positive tumor with a sclerosing epithelioid fibrosarcoma-like pattern, further expanding the morphological spectrum of NTRK-RSCNs.

Zhang et el (4). reported a case in which a primary tumor with an LPF-NT-like pattern progressed to a malignant tumor with an MPNST-like pattern at recurrence, suggesting that NTRK-RSCNs can present low-grade to high-grade morphologic transformations during disease progression. We need to focus on the differences in clinicopathologic features before and after recurrence to refine the treatment plan for patients. Meanwhile, NTRK-RSCNs showed heterogeneous differentiation. Tsai reported two NTRK-RSCNs cases in which a fibrosarcoma-like uniform spindle cell component abruptly transformed a pleomorphic liposarcoma in a cervical tumor, and a pleural tumor harbored scattered heterologous rhabdomyoblasts in an MPNST-like background (5). We need to recognize the heterogeneity of NTRK-RSCNs histological patterns and help further diagnosis with the support of immunohistochemistry and molecular testing.

Immunohistochemical co-expression of CD34 and S100 protein is a highly important diagnostic clue when the histologic pattern is not specific. Nearly 70% of our summarized cases presented CD34 and S100 co-expression, but there were still 22.8% of cases showing CD34-/S100+ (12.6%) or CD34+/S100- (10.2%), and even 7.9% of cases showed CD34-/S100-. This may lead to a great challenge for the diagnosis of NTRK-RSCNs and makes it very easy to miss and misdiagnose. Also, the two groups showed different preferences for single positive CD34/S100 expression. Compared with NTRK-RSCNs in soft tissues, visceral NTRK-RSCNs showed a higher frequency of CD34-/S100+ immunophenotype.

In addition, the aforementioned morphological features, along with the CD34+/S100+ immunophenotype, do not seem to be unique to NTRK-RSCN. Overlapping features have been observed in tumors harboring diverse protein kinase fusions, particularly those involving RET, RAF1, and BRAF (30). Immunohistochemistry is highly sensitive to pan-TRK, which is crucial for the diagnosis of NTRK-RSCNs of CD34-/S100+, CD34+/S100-, and CD34-/S100-, as well as to exclude tumors fused with other kinases in the presence of co-expression of CD34 and S100. Pan-TRK is an antibody against TRKA, TRKB, and TRKC proteins. Immunohistochemistry of pan-TRK shows distinct positive expression patterns in different NTRK gene fusions. Immunohistochemistry of pan-TRK often demonstrates cytoplasmic staining in NTRK tumors with NTRK1/2 gene fusions and nuclear staining with or without weak cytoplasmic staining in NTRK tumors with NTRK3 gene fusion. The sensitivity of pan-TRK immunohistochemistry was higher for detecting NTRK1 (96%) and NTRK2 (100%), while NTRK3 fusion sensitivity was only 79%, including weak and focal staining (less than 5% of cells). Therefore, any pan-TRK positive staining in at least 1% of tumor cells was classified as positive (51). In our data, 99% of cases pan-TRK IHC exhibited positive staining, of which 13.9% of cases pan-TRK IHC showed focal staining. Although pan-TRK immunohistochemistry serves as a crucial screening tool, its utility might be limited by non-fusion-induced NTRK overexpression in certain tissue/tumor types. Tsai et al. performed pan-TRK immunohistochemistry on 278 mesenchymal tumors, which could serve as a reasonable differential diagnosis of visceral NTRK-RSCNs. The results revealed that 56% of BCOR-positive sarcomas, 50% of undifferentiated uterine sarcomas, and 33% of spindle cell/sclerosing rhabdomyosarcomas presented moderate-intensity staining ranging from 0 to 56% (7). The overall sensitivity of pan-TRK antibodies was approximately 85–90%; however, the overall specificity was well less than 50% (5, 8). We should be aware of the limitation of pan-TRK immunostaining and seek molecular corroboration when the specificity of pan-TRK staining regarding the histotypes in consideration is unsatisfactory. Besides, some cases were positive for SMA (3, 52), CD99 (41), and CD30 (9). Meanwhile, Ultrastructural analyses evidenced a myofibroblastic differentiation in NTRK-RSCNs (12). In NTRK-RSCNs, we usually observed SOX10 negativity and H3K27me3 expression retention, which helped to discriminate from MPNST.

Olson et al. (43) reported an aggressive sarcoma in the primary tumor was immunohistochemically CD34 positive and diagnosed as DSFP with fibrosarcomatous transformation. One year later, the patient suffered a recurrence and the recurrent sample was immunohistochemically negative for S-100 and CD34, negative for SOX-10, and completely absent for H3K27Me3. Subsequent additional pan-TRK immunohistochemistry showed weak cytoplasmic staining, but further molecular testing and targeted therapy were not done and the patient developed metastasis after 5 years. The presence of EML4-NTRK3 fusion was confirmed for recurrent and metastatic tumors using RT-PCR followed by Sanger and RNA sequencing. The histologic diversity of NTRK-RSCNs makes it a great diagnostic challenge, and as a result, the risk of missing effective treatment is high. Pathologists should be equipped with a high level of awareness and useful diagnostic aids, recognize the importance and limitations of pan-TRK immunohistochemistry, and ultimately rely on molecular assays to reach a precise diagnosis.

Fluorescence *in situ* hybridization (FISH) is fast and economical. The incidence of NTRK1 rearrangements (68.9%, 111/161) was much higher than in NTRK2 (1.9%, 3/161) and NTRK3 (29.2%, 47/161) rearrangements, consistent with the literature (53). NTRK1 gene fusion rate was remarkably higher in soft tissues than in viscera. TPM3::NTRK1 fusion was the most common translocation, and the other NTRK1 fusion partners included LMNA, TPR, IRF2BP2, TMB3, and C16orf72. However, the NTRK3 gene fusion rate was significantly higher in viscera than in soft tissues, the most common partner gene was EVT6, and the remaining fusion partners included SPECC1L, EML4, STRN, TFG, SQSTM1, BPMS, and RBPMS. Only three cases (1.9%, 3/161) had NTRK2 mutations (fusion partners were identified, SPECC1L, STRN, and WWOX, respectively). With the increasing number of reports, more NTRK fusion genes will be discovered, and the gene spectrum of NTRK-RSCNs will be more comprehensive. However, FISH not only fails to provide information about the fusion partners but may also produce false-negative results on account of atypical small-gap or unbalanced split signals. RT-PCR is a high-sensitivity method, but it is only suitable for detecting known fusions and cannot detect multiple NTRK gene fusions at the same time (19). NGS is ideal for tumor types with low incidence of NTRK fusion and can identify new tumor types. There are two types of NGS: DNA sequencing and RNA sequencing. RNA-NGS has higher sensitivity than DNA-NGS and avoids technical problems caused by intronic regions (54). It is suggested that both DNA and RNA testing be performed when conditions permit to improve the detection rate and accuracy.

In this patient, genome sequencing identified a significant fusion mutation EVT6-NTRK3 with 35.17% mutation abundance. Besides, this NTRK-RSCNs case showed CDKN2A/B deletion and ARID1A mutation. In our series, loss of the tumor suppressors CDKN2A and CDKN2B occurred frequently (77.4%, 24/31). CDKN2A/CDKN2B DNA copy number aberrations have been reported to be highly prevalent in MPNST, myxofibrosarcoma, and undifferentiated pleomorphic sarcomas and were associated with a poor prognosis in soft tissue sarcomas. This phenomenon challenges the concept that NTRK fusions were mutually exclusive from other oncogenic drivers (5). The ARID1A gene is a tumor suppressor encoding the ARID1A protein, whose inactivating mutation is an essential element in the development of many types of tumors, including ovarian, breast, and renal cancers etc (55). In previous studies, the NTRK gene fusion had also been accompanied by some rare gene mutations, including FOXL2 mutation, SMAD4 and CHEK2 deletion, and MCL1 and MYC gene copy number increases (4). Pathologists should be aware of these genomic co-alterations to improve the best methods for NTRK gene fusion screening.

NTRK-RSCNs should be differentially diagnosed from other diseases as follows: (1) RET, MET, RAF1, BRAF, ALK kinase fusion tumors: histological morphology was similar to that of NTRK-RSCNs, and immunohistochemistry also showed co-expression of S100 and CD34. However, pan-TRK was negatively expressed and NTRK rearrangement was not detected by molecular testing (30). (2) inflammatory myofibroblastic tumor: immunohistochemistry α-SMA, Desmin, and ALK were positive, and both CD34 and S100 proteins were negative. ALK protein expression and gene rearrangement contributed to the differentiation of the two tumors (4). (3) MPNST: SOX10 positive and H3K27me3 loss were helpful in differentiation (4). (4) solitary fibrous tumor (SFT): immunohistochemistry STAT6 was positive and molecular testing confirmed NAB2-STAT6 fusion (56, 57). (5) synovial sarcoma: tumor cells express CAM5.2, EMA, bcl-2, CD99, and Calponin, and molecular testing showed the SS18 gene translocation (58). (6) gastrointestinal stromal tumor (GISTs): GISTs showed diffuse and strong expression of CD117 and/or DOG1, which could help to distinguish them from NTRK-RSCNs (4). (7) sclerosing epithelioid fibrosarcoma: tumor cells expressed MUC4 and did not express CD34 and S100 (59).

Surgical resection is the predominant therapy for NTRK-RSCNs, and most patients with complete surgical resection have a better prognosis. Of the 30 cases with recurrence and metastasis, 8 cases were observed with CDKN2A/2B copy number deletion, 3 cases with positive surgical margins, and 1 case with H3K27Me3 deletion (2, 43). This suggests that the prognosis of the tumor was likely to be associated with positive surgical margins and genomic co-alterations such as CDKN2A/2B deletions. Of these 30 cases, there were 7 cases of LPF-NT-like morphology, 6 cases of MPNST/fibrosarcoma-like morphology, 1 case of adenosarcoma-like morphology, 1 case of adenosarcoma-like morphology, and 1 case of IMT-like morphology (2, 4, 5, 17, 19, 24, 33, 37, 41). Therefore, the relevance of histologic morphologic heterogeneity and prognosis of NTRK-RSCNs needs to be further verified in a large number of cases.

For patients who have difficulty with complete surgical resection and postoperative metastasis/recurrence, adjuvant treatment with NTRK inhibitors, such as larorectinib, and entrectinib, is recommended. Therefore, pathologists should indicate histologic grading and margins in the pathological report, suggesting clinical prognosis and further choice of treatment. This patient was additionally treated with the NTRK inhibitor larorectinib after surgery. A follow-up of 22 months demonstrated no evidence of local recurrence or metastatic disease.

Larotrectinib and entrectinib are orally available first-generation TRK inhibitors (54). Both are type I inhibitors that bind the active conformation (xDFG-in) of TRK kinases, competing with the endogenous substrate for the ATP binding site (60). Both produce robust anti-tumor efficacy regardless of tumor type and NTRK fusion type (61).

Larotrectinib is the first targeted agent in the world to be used for initial treatment regardless of tumor source. In 2018, the United States Food and Drug Administration (FDA) approved larotrectinib, for the treatment of NTRK gene fusion tumors in both adult and pediatric populations. In 2023, Drilon et al. updated the results of a prespecified combined analysis of three clinical trials evaluating the activity of larotrectinib in patients with locally advanced or metastatic NTRK fusion-positive solid tumors (62). Among the 289 patients enrolled, the objective response rate was 69% (95% CI, 60–72%), with a complete response rate of 27%. The median progression-free survival was 30.8 months (95% CI, 19.3–34.3 months) after a median follow-up of 31.3 months (62, 63).

Different from larotrectinib, entrectinib halts reactive oxygen species oncogene 1 (ROS1) and anaplastic lymphoma kinase (ALK) in addition to blocking tyrosine receptor kinase A, B, and C [31]. In 2019 and 2020, entrectinib received United States and European Union approval/marketing authorizations for the treatment of patients ≥12 years old with NTRK fusion-positive solid tumors and adults with ROS1 fusion-positive non–small cell lung cancer (NSCLC) (64). In 2022, Demetri et al. updated the results of an integrated analysis of three previous phase I/II trials (ALKA-372-001; STARTRK-1; STARTRK-2) evaluating 121 patients with advanced or metastatic solid tumors. The investigator-assessed partial response rate was 45.4%, with a complete response rate of 15.7%. The median progression-free survival was 13.8 months (95% CI, 10.1–19.9 months) after a median follow-up of 25.8 months. In addition, entrectinib is a weak substrate for P-gp, penetrates the blood-brain barrier better than larotrectinib, reaches effective concentrations in the CNS, and maintains good activity. Entrectinib has demonstrated superior intracranial efficacy benefits in several clinical studies (65).

It is not appropriate to directly use a simple side-by-side comparison to compare the ORR of the two drugs because the types and number of tumors studied are not fully aligned. Jesus Garcia-Foncillas et al. evaluated the differences in efficacy and safety between larotrectinib and entrectinib using the matching-adjusted indirect comparison (MAIC) method (66). The results showed that the median OS of larotrectinib and entrectinib in treating patients with NTRK gene fusion tumors was not reached at 23.9 months, respectively (95% CI:0.23-0.83, *P*<0.05), so larotrectinib was associated with significantly longer OS compared with entrectinib. Both had similar ORRs (67.3% vs. 63.5%; *P* = 0.63), but larotrectinib had the higher complete response rate (20.3% vs. 6.8%; *P* < 0.05) and longer DOR (median 32.5 vs. 12.9 months; *P* < 0.05) (67).

Although first-generation NTRK inhibitors offer significant clinical benefits to tumor patients, the development of drug resistance remains a challenge. The mechanisms of drug resistance can be classified into two types: on-target and off-target mechanisms (68). The development of second-generation TRK inhibitors aims to overcome these resistance mechanisms and offer novel therapeutic options for patients experiencing disease relapse or resistance to first-generation inhibitors (69). Repotrectinib, selitrectinib, and taletrectinib represent the leading second-generation TRK inhibitors in clinical development (54). Based on phase 1/2 data from the TRIDENT-1 study, the U.S. FDA granted accelerated approval to repotrectinib in 2023 for advanced solid tumors harboring NTRK gene fusions. Clinical trials evaluating selitrectinib and taletrectinib are ongoing, with mature efficacy and safety data pending publication (70).

The use of these pan-TRK inhibitors may lead to treatment-related side effects due to the inhibition of TRK signaling in normal tissues (71, 72). Notably, most treatment-related adverse events were categorized as grade 1–2, such as dizziness, constipation, ataxia, balance disorder, and dysgeusia, with only a small fraction exhibiting grade 3–4 severity, including myalgia, hypersensitivity reactions, and weight gain (48, 68).

In summary, we reported the first case of primary pancreas NTRK-rearranged spindle cell tumor with a special sclerosing epithelioid fibrosarcoma pattern harboring EVT6::NTRK3 gene fusions, CDKN2A/2B homozygous deletion, and ARID1A mutation. Reviewing the literature, we found that there may be differences in age, histomorphology, immunophenotype, genetics, and prognosis between visceral and soft tissue NTRK-RSCNs. The visceral NTRK-RSCNs were not uncommon, mostly in the uterus and gastrointestinal tract, and were prevalent in young and middle-aged people. The visceral NTRK-RSCNs mainly exhibited high-grade morphology such as MPNST/fibrosarcoma-like, characterized by co-expression of CD34, S100, and pan-TRK. The visceral NTRK-RSCNs showed a higher frequency of NTRK3 fusion, a lower frequency of NTRK1 fusion, and a higher mortality rate. Due to the nonspecific and highly variable morphology, the diagnosis of these tumors is difficult, leading to hence a high risk of missing out on effective treatment. Thus, pathologists should be possessed of a high awareness of NTRK-RSCNs. Appropriate immunohistochemical workup, including CD34, S100, and pan-TRK as a screening tool and molecular tests, are indispensable in identifying this entity.

## Data Availability

The original contributions presented in the study are included in the article/**Supplementary Material**. Further inquiries can be directed to the corresponding author.
