# Supplementary figures and images for: Primary pancreas NTRK-rearranged neoplasm harboring an EVT6::NTRK3 fusion with a sclerosing epithelioid fibrosarcoma morphology: a case report and comprehensive review of the literature
Source: Front Oncol. 2025 Jun 6;15:1526281. doi: 10.3389/fonc.2025.1526281 (PMC12178828; doi:10.3389/fonc.2025.1526281)

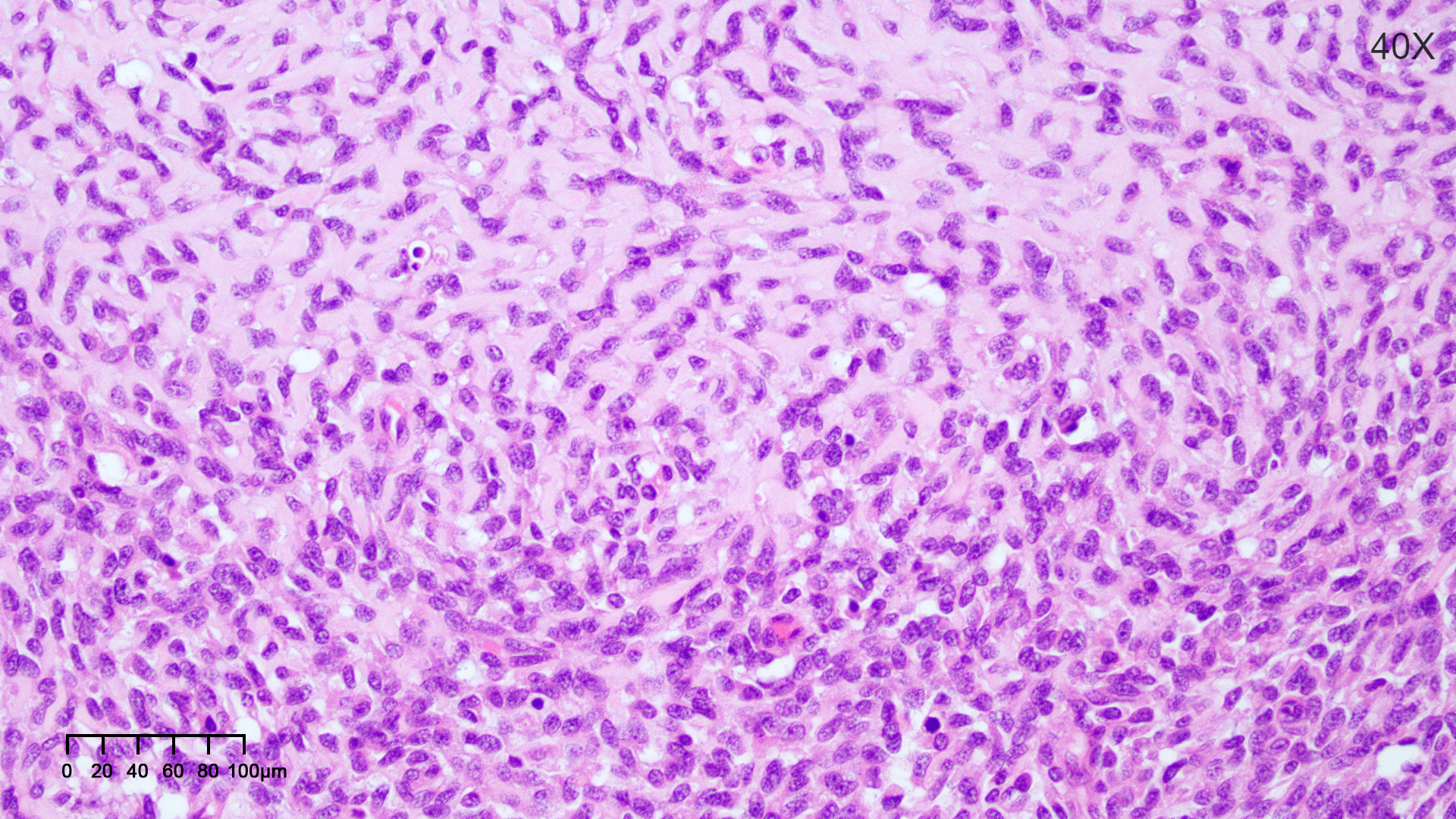

Supplement: Supplementary file 1 [file Image1.tif]

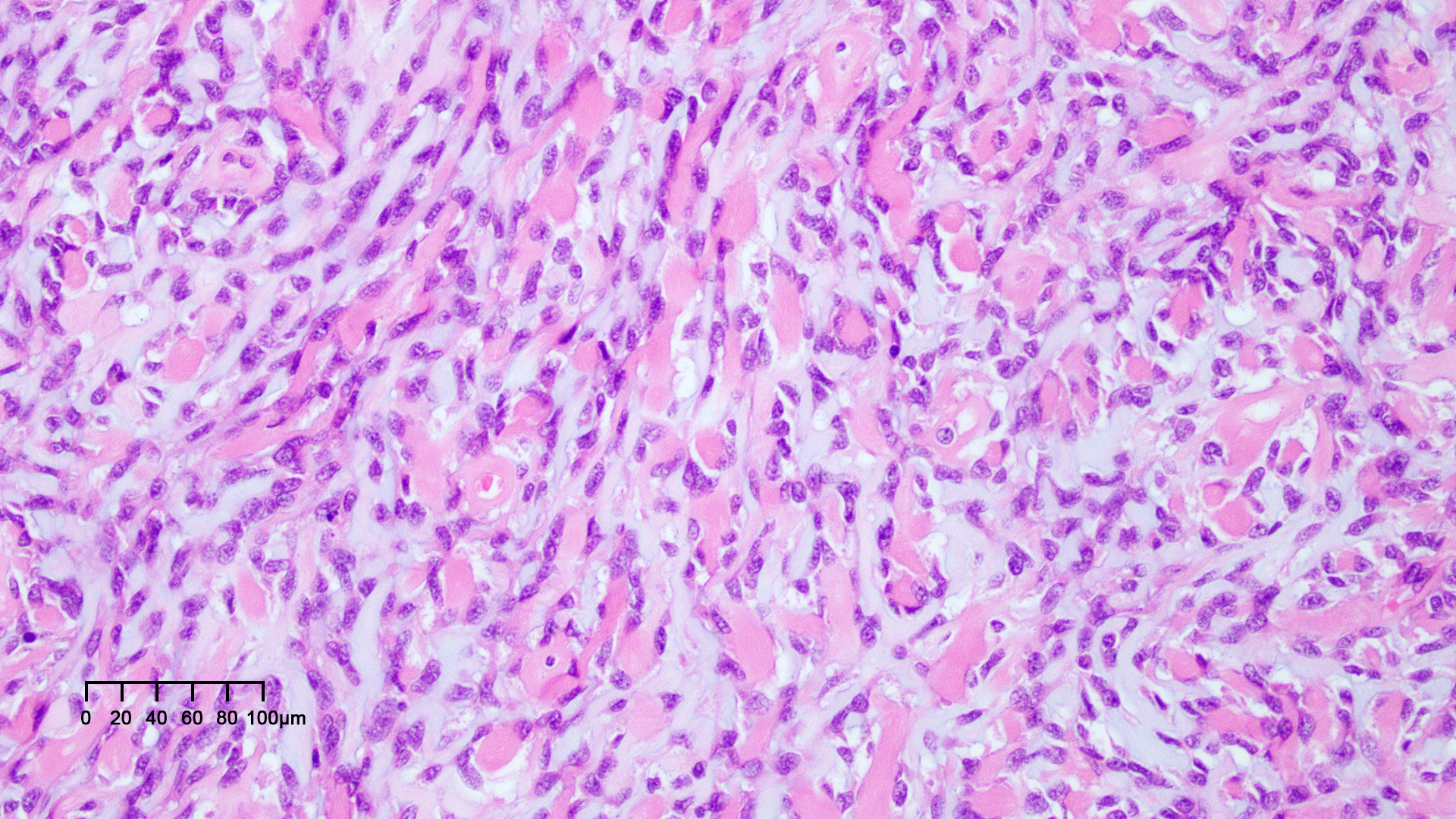

Supplement: Supplementary file 2 [file Image2.tif]

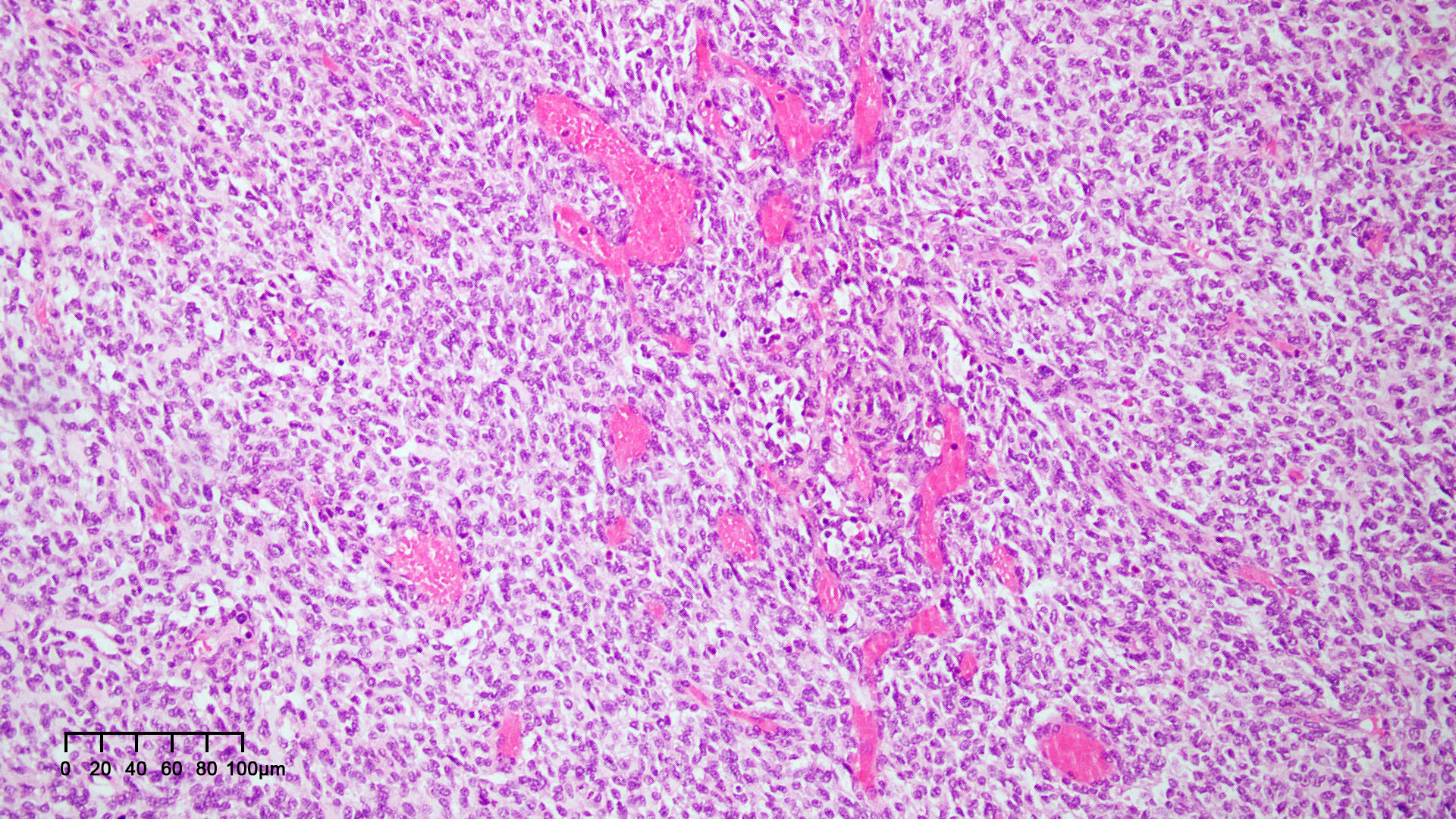

Supplement: Supplementary file 3 [file Image3.tif]

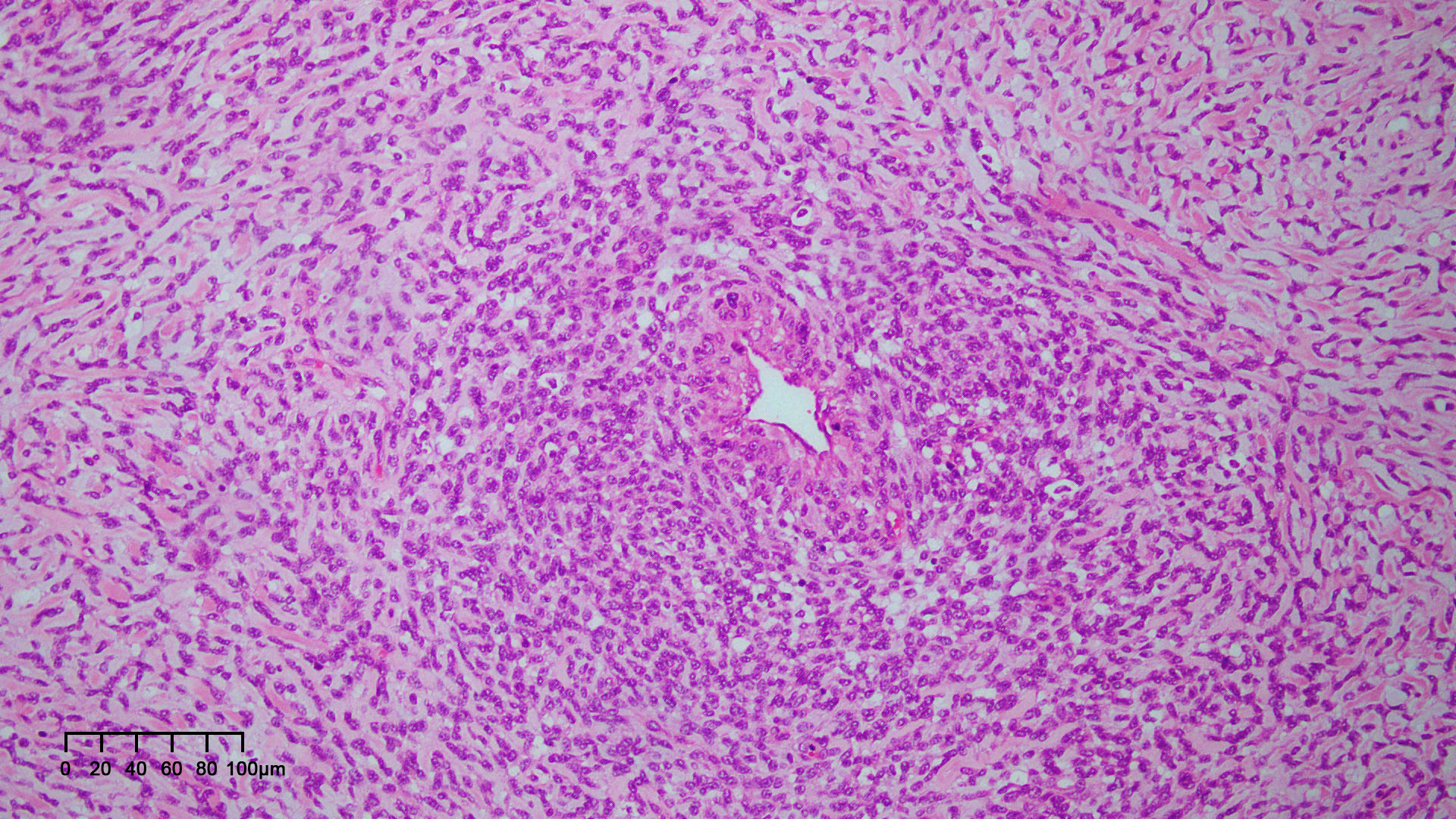

Supplement: Supplementary file 4 [file Image4.tif]

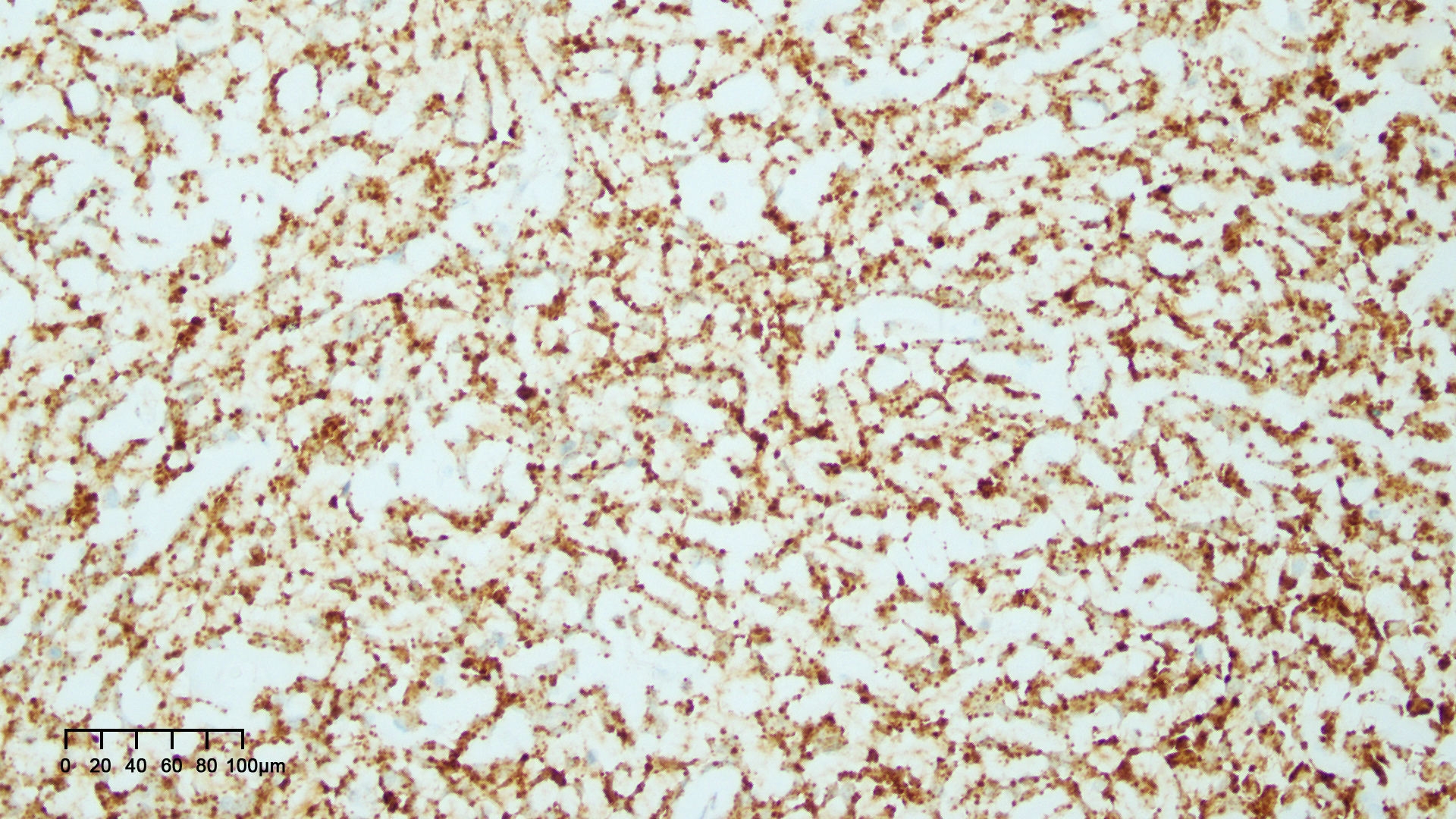

Supplement: Supplementary file 5 [file Image5.tif]

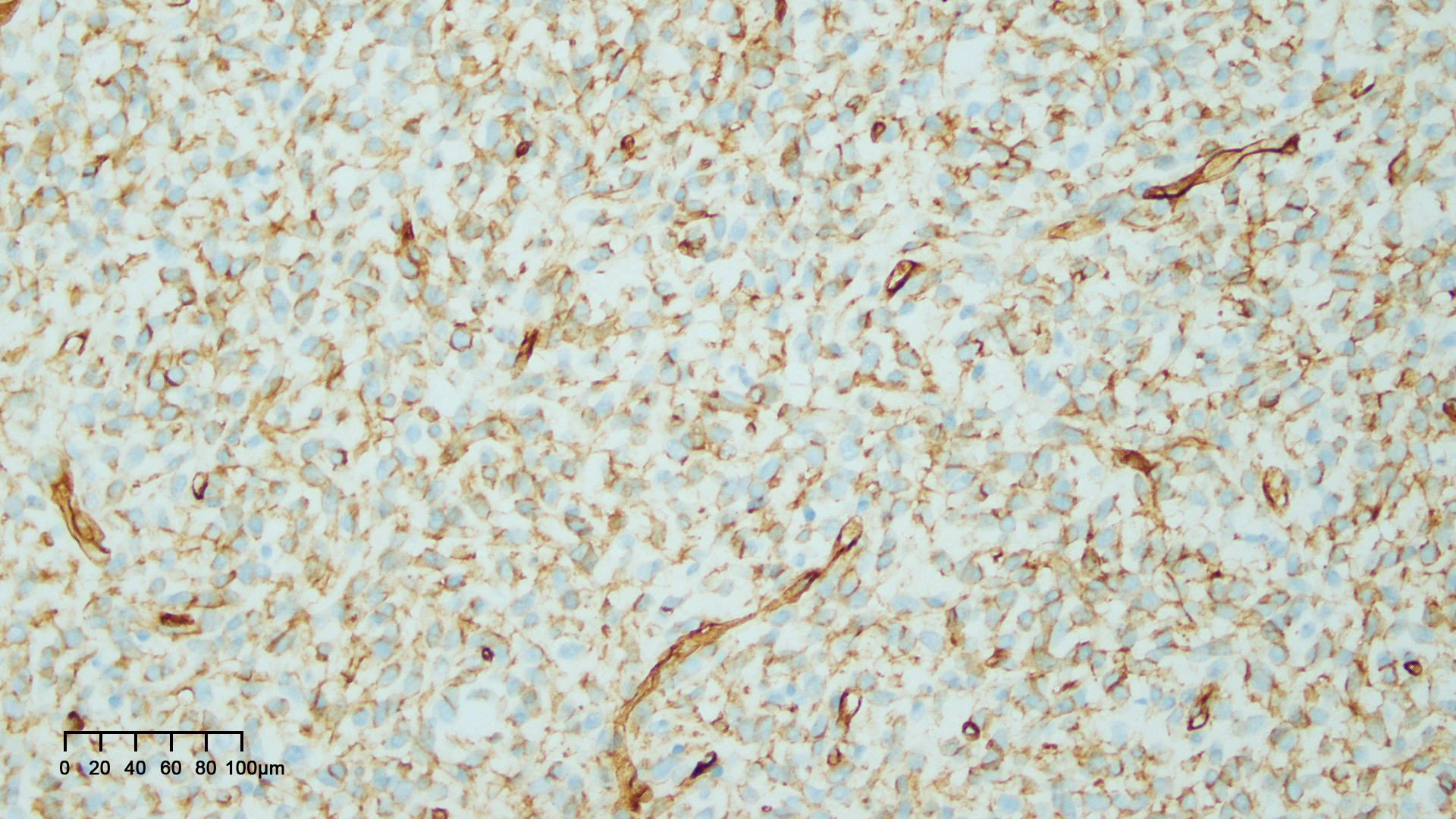

Supplement: Supplementary file 6 [file Image6.tif]

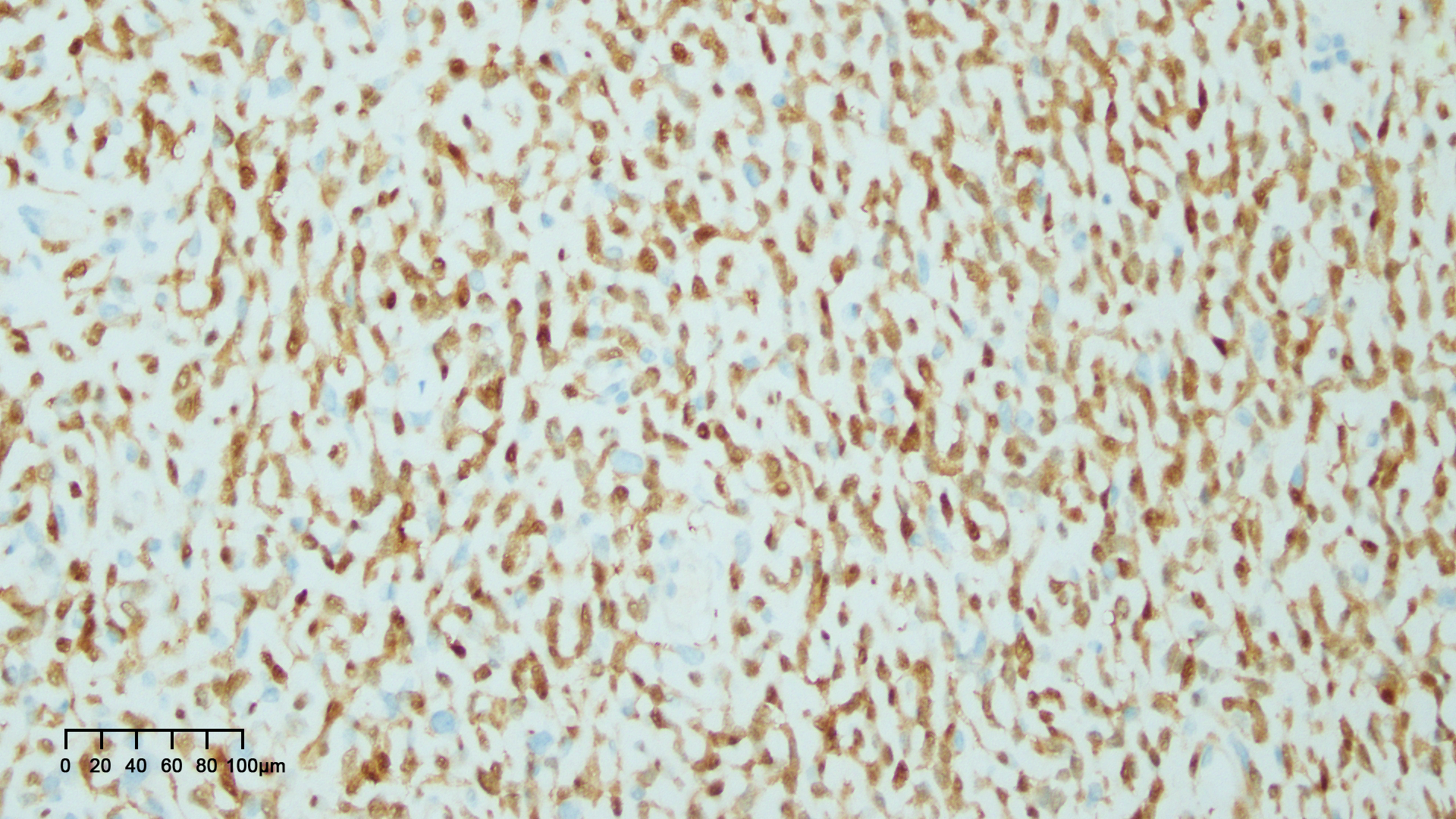

Supplement: Supplementary file 7 [file Image7.tif]
